# Sex-based outcomes on unguided de-escalation from ticagrelor to clopidogrel in stabilized patients with acute myocardial infarction undergoing percutaneous coronary intervention: a *post-hoc* analysis of the TALOS-AMI

**DOI:** 10.3389/fcvm.2024.1358657

**Published:** 2024-03-21

**Authors:** Eun-Seok Shin, Eun Jung Jun, Bitna Kim, Chan Joon Kim, Mahn-Won Park, Eun-Ho Choo, Byung-Hee Hwang, Kwan Yong Lee, Gyu-Chul Oh, Min Chul Kim, Hyeon Woo Yim, Youngkeun Ahn, Kiyuk Chang

**Affiliations:** ^1^ Department of Cardiology, Ulsan University Hospital, University of Ulsan College of Medicine, Ulsan, Republic of Korea; ^2^ Division of Cardiology, Department of Internal Medicine, Uijeongbu St. Mary’s Hospital, The Catholic University of Korea, Uijeongbu, Republic of Korea; ^3^ Division of Cardiology, Department of Internal Medicine, Daejeon St. Mary’s Hospital, The Catholic University of Korea, Daejeon, Republic of Korea; ^4^ Department of Internal Medicine, Division of Cardiology, Seoul St. Mary’s Hospital, The Catholic University of Korea, Seoul, Republic of Korea; ^5^ Department of Cardiology, Chonnam National University Hospital, Chonnam National University Medical School, Chonnam, Republic of Korea; ^6^ Department of Preventive Medicine, College of Medicine, The Catholic University of Korea, Seoul, Republic of Korea

**Keywords:** sex, acute myocardial infarction, clopidogrel, ticagrelor, percutaneous coronary intervention, drug-eluting stent

## Abstract

**Background:**

The TALOS-AMI study highlighted the effectiveness of a de-escalation strategy shifting from ticagrelor to clopidogrel 1 month after percutaneous coronary intervention (PCI), resulting in significant reduction in clinical events, primarily attributed to a substantial decrease in bleeding events. Nevertheless, the impact of this strategy on outcomes based on sex remains unclear.

**Methods:**

This was a *post-hoc* analysis of the TALOS-AMI study. At 1 month after PCI, patients who remained adherent to aspirin and ticagrelor without experiencing major adverse events were randomized into either the de-escalation group (clopidogrel plus aspirin) or the active control group (ticagrelor plus aspirin) for an additional 12 months. The primary endpoint encompassed a composite of cardiovascular death, myocardial infarction, stroke, and Bleeding Academic Research Consortium bleeding type 2 or greater at 12 months after randomization.

**Results:**

Among the 2,697 patients included in this study, 454 (16.8%) were women. Women, characterized by older age and a higher prevalence of hypertension, diabetes, impaired renal function, and non-ST-segment myocardial infarction, exhibited a lower primary endpoint at 12 months compared to men [adjusted hazards ratio (HR), 0.60; 95% confidence interval (CI), 0.37–0.95; *P* = 0.03]. Compare to the active control group, the de-escalation group demonstrated a reduced risk of the primary endpoint in both women (adjusted HR, 0.38; 95% CI, 0.15–0.95; *P* = 0.039) and men (adjusted HR, 0.56; 95% CI, 0.40–0.79; *P* = 0.001) (interaction *P* = 0.46).

**Conclusions:**

In stabilized patients post-PCI with drug-eluting stents for acute myocardial infarction, the primary endpoint was lower among women compared to men. In this cohort, the benefits of an unguided de-escalation strategy from ticagrelor to clopidogrel were comparable in women and men.

## Introduction

The current guidelines recommend prioritizing potent P2Y12 receptor inhibitors over clopidogrel for up to 1 year in patients with acute myocardial infarction (AMI) undergoing percutaneous coronary intervention (PCI) with drug-eluting stents (DESs) (1). While the ischemic risk is more pronounced in the early phase, the bleeding risk remains high during the AMI maintenance phase. These findings have led to a stepwise de-escalation approach of dual antiplatelet therapy (DAPT), utilizing a potent P2Y12 inhibitor in the acute phase and transitioning to the less potent clopidogrel during the chronic phase of treatment.

Recently, findings from the Ticagrelor vs. Clopidogrel in Stabilized Patients with AMI (TALOS-AMI) study have demonstrated that an unguided de-escalation strategy significantly reduces the risk of net clinical events up to 12 months in stabilized patients with AMI after PCI with DES. The reduction is primarily attributed to decreased bleeding events (2). However, it remains uncertain whether these effects vary based on sex. Despite women having an increased risk of both ischemic and bleeding events after PCI compared with men (3–6), it is unknown whether women face an elevated risk of these events in stabilized patients with AMI after PCI. Therefore, a *post-hoc* analysis was conducted to assess outcomes by sex in stabilized patients with AMI who underwent PCI with DES and were enrolled in the TALOS-AMI study.

## Materials and methods

### Study design

This was a *post-hoc* analysis of the TALOS-AMI study. The TALOS­AMI study was initiated by an investigator and conducted as a prospective, open­label, multicenter, randomized study (2, 7). Enrollment occurred at 32 institutes in South Korea. The study protocol received approval from the institutional review board at each participating institute, and all participants provided written informed consent. External oversight for participant safety was provided by an independent data and safety monitoring board. An independent clinical event adjudication committee (CEAC), whose members were blinded to the trial group, was responsible for adjudicating all events. The CEAC members reviewed medical records of adverse events after removing any reference to the treatment group. The TALOS-AMI study took place from 26 February 2014 to 31 December 2018.

### Study population and study regimen

To meet enrollment criteria, patients had to undergo successful PCI with a current generation DES and tolerate aspirin and ticagrelor treatment during the index admission. Screening for eligibility involved assessing the completion of aspirin and ticagrelor treatment without major adverse ischemic events [myocardial infarction (MI), stroke, or unplanned revascularization] or bleeding events at 1 month after PCI. Random assignment of selected patients was then carried out, with individuals being allocated to either the aspirin plus ticagrelor group or the aspirin plus clopidogrel group for a duration of 12 months. Key exclusion criteria encompassed cardiogenic shock, active bleeding from any major organs, bleeding diathesis or coagulopathy, gastrointestinal or genitourinary bleeding, hemoptysis, a history of intracranial bleeding, intracranial aneurysm, arteriovenous malformation, and neoplasm.

All participants received a ticagrelor loading dose (180 mg), and those not naïve to aspirin were administered an aspirin loading dose (250–325 mg) before PCI. Subsequently, they were given ticagrelor 90 mg twice daily and aspirin 100 mg daily for the following 30 days. At 30 ± 7 days after PCI, eligible patients were randomly assigned in a 1:1 ratio to either continue ticagrelor (active control group) or switch to clopidogrel 75 mg daily without a loading dose (de-escalation group). The de-escalation group was conducted without guidance from genetic or platelet function testing.

### Outcomes

The primary outcome comprised a combination of cardiovascular death, MI, stroke, and bleeding events categorized as type 2, 3, or 5 according to the Bleeding Academic Research Consortium (BARC) criteria (8). These events occurred between 1 and 12 months after the index PCI. The bleeding endpoint involved a combination of BARC bleeding types 2, 3, or 5. Comprehensive definitions for each clinical event have been previously provided (2). The ischemic endpoint constituted a combination of cardiovascular death, MI, stroke, any revascularization, or stent thrombosis.

### Statistical analysis

The baseline clinical and procedural characteristics were stratified based on sex and the randomized treatment group. Continuous variables were presented as means and standard deviations, while categorical variables were expressed as frequencies and percentages. The primary analysis for the primary endpoint, bleeding, and ischemic events was conducted within the intention-to-treat population. The cumulative incidences of primary endpoints were estimated using the Kaplan–Meier method. Patients without a primary endpoint event between randomization and 2 years were censored at the time of death, last known contact, or 12 months, whichever occurred first.

Hazard ratios (HRs) and 95% confidence intervals (CIs) were calculated using Cox proportional hazards models. Deaths were categorized into cardiac, vascular, and non-cardiovascular causes. Cardiac death included any death resulting from a proximate cardiac cause (e.g., MI, low-output failure, fatal arrhythmia), unwitnessed death, and death of unknown cause, including all procedure-related deaths, which were classified as cardiac death. Vascular death was defined as death caused by non-coronary vascular factors, such as cerebrovascular disease, pulmonary embolism, ruptured aortic aneurysm, dissecting aneurysm, or other non-coronary causes. Non-cardiovascular death encompassed any deaths not covered by the above definitions, such as those caused by infection, malignancy, sepsis, pulmonary causes, accident, suicide, or trauma.

Associations of sex with the outcomes were examined using Cox regression. Models were adjusted for variables that displayed baseline differences, including age, body mass index, hypertension, diabetes, current smoking, impaired renal function, AMI, access site, glycoprotein IIb/IIIa inhibitor, number of stents, total stent length, and mean stent diameter. Treatment outcomes of the de-escalation strategy vs. the active control group were evaluated by sex, and formal interaction testing using Cox regression was performed to assess for effect modification. An interaction test was used to determine whether the relative effects of the study treatments varied significantly between subgroups. Participants with missing primary and secondary endpoint data were censored at the time of consent withdrawal or loss to follow-up. *P*-values were two-sided, and statistical significance was set at *P* < 0.05. All statistical analyses were conducted using R (version 4.1.2; R Foundation for Statistical Computing, Vienna, Austria). Data were analyzed for the period from October to December 2021. This study is registered with ClinicalTrials.gov, NCT02018055.

## Results

### Baseline clinical and procedural characteristics

A total of 2,901 patients were enrolled, and 2,697 underwent randomization. Among the randomized patients, 454 (16.8%) were women, with a mean (SD) age of 60.0 (11.4) years. Table 1 presents the baseline clinical and procedural characteristics by sex. In comparison to men, women were older [mean (SD) age, 68.0 (10.1) years vs. 58.3 (10.9) years] and had a higher prevalence of hypertension, diabetes, and impaired renal function [82 women (18.1%) vs. 223 men (9.9%)]. Conversely, women had a lower body mass index and were less likely to be current smokers (Table 1). Compared to men, women were less likely to have undergone PCI for an indication of ST-segment elevation MI and glycoprotein IIb/IIIa inhibitor use, but they were more likely to have used radial access. No significant differences were noted regarding the culprit vessel location, the number of treated vessels, and the extent of coronary artery disease. However, women had fewer stents used, longer stent lengths, and smaller stent diameters compared to men (Table 1).

**Table 1:** Baseline clinical and procedural characteristics by sex.

	No. (%)		*P*-value
	Women (*n* = 454)	Men (*n* = 2,243)	
Age, mean (SD), years	68.0 (10.1)	58.3 (10.9)	**<0** **.** **001**
≥75	133 (29.3)	188 (8.4)	**<0**.**001**
Body mass index, mean (SD), kg/m^2^	23.9 (3.5)	24.7 (3.0)	**<0**.**001**
Cardiovascular risk factors			
Hypertension	280 (61.7)	1,038 (46.3)	**<0**.**001**
Diabetes mellitus	154 (33.9)	577 (25.7)	**<0**.**001**
Diabetes treated with insulin	14 (3.1)	42 (1.9)	0.142
Dyslipidemia	196 (43.2)	923 (41.2)	0.461
Current smoker	70 (15.4)	1,274 (56.8)	**<0**.**001**
Impaired renal function	82 (18.1)	223 (9.9)	**<0**.**001**
Past medical history			
Previous percutaneous coronary intervention	22 (4.8)	99 (4.4)	0.780
Previous coronary artery bypass graft	2 (0.4)	2 (0.1)	0.269
Previous cerebrovascular accident	25 (5.5)	78 (3.5)	0.055
Clinical presentation			
STEMI	196 (43.2)	1,259 (56.1)	**<0**.**001**
NSTEMI	258 (56.8)	984 (43.9)	**<0**.**001**
Left ventricular ejection fraction <40%	28 (6.2)	168 (7.5)	0.400
Access site			
Radial	253 (55.7)	1,099 (49.0)	**0**.**010**
Femoral	193 (42.5)	1,118 (49.8)	**0**.**005**
Glycoprotein IIb–IIIa inhibitor	86 (18.9)	558 (24.9)	**0**.**008**
Infarct-related artery (culprit)			
Left main	8 (1.8)	37 (1.6)	>0.999
Left anterior descending	229 (50.4)	1,090 (48.6)	0.506
Left circumflex	78 (17.2)	388 (17.3)	>0.999
Right coronary	139 (30.6)	725 (32.3)	0.512
Number of treated vessels, mean (SD), No	1.3 (0.6)	1.4 (0.6)	0.322
Multivessel treatment			
2 vessels	108 (23.8)	514 (22.9)	0.733
3 vessels	26 (5.7)	106 (4.7)	0.434
Number of stents for infarct-related artery, mean (SD), No	1.17 (0.41)	1.23 (0.45)	**0**.**006**
Total stent length of the infarct-related artery, mean (SD), mm	31.7 (14.8)	29.3 (13.2)	**0**.**001**
Stent diameter of the infarct-related artery, mean (SD), mm	3.0 (0.4)	3.2 (0.5)	**<0**.**001**
Optical coherence tomography	9 (2.0)	73 (3.3)	0.197
Intravascular ultrasonography	100 (22.0)	540 (24.1)	0.388

NSTEMI, non-ST-segment elevation myocardial infarction; STEMI, ST-segment elevation myocardial infarction.

The body mass index is the weight in kilograms divided by the square of the height in meters. Impaired renal function was defined as an estimated glomerular filtration rate of less than 60 ml/min per 1.73 m^2^ of body surface area at presentation.

Bold type *p* value, there are statistically significant differences at the significance level of 5%.

Table 2 presents the baseline clinical and procedural characteristics according to sex and the randomized treatment group. Among women, baseline clinical characteristics were well-balanced between the treatment groups. Among men, patients randomized to the active control group were more likely to have lesions in the left circumflex artery compared to those randomized to the de-escalation group (Table 2).

**Table 2:** Baseline clinical and procedural characteristics by sex and randomized treatment assignment.

	Women (*n* = 454), No. (%)			Men (*n* = 2,243), No. (%)		
	De-escalation (*n* = 217)	Active control (*n* = 237)	*P*-value	De-escalation (*n* = 1,132)	Active control (*n* = 1,111)	*P*-value
Age, mean (SD), years	68.0 (10.3)	68.0 (10.0)	0.980	58.5 (10.8)	58.2 (11.0)	0.404
≥75	66 (30.4)	67 (28.3)	0.690	91 (8.0)	97 (8.7)	0.606
Body mass index, mean (SD), kg/m^2^	24.0 (3.5)	23.7 (3.4)	0.395	24.8 (3.0)	24.7 (3.1)	0.694
Cardiovascular risk factors						
Hypertension	139 (64.1)	141 (59.5)	0.367	516 (45.6)	522 (47.0)	0.520
Diabetes mellitus	72 (33.2)	82 (34.6)	0.826	290 (25.6)	287 (25.8)	0.936
Diabetes treated with insulin	10 (4.6)	4 (1.7)	0.127	18 (1.6)	24 (2.2)	0.401
Dyslipidemia	93 (42.9)	103 (43.5)	0.972	470 (41.5)	453 (40.8)	0.766
Current smoker	39 (18.0)	31 (13.1)	0.190	631 (55.7)	643 (57.9)	0.316
Impaired renal function	43 (19.8)	39 (16.5)	0.469	117 (10.3)	106 (9.5)	0.535
Past medical history						
Previous percutaneous coronary intervention	7 (3.2)	15 (6.3)	0.187	54 (4.8)	45 (4.1)	0.470
Previous coronary artery bypass graft	1 (0.5)	1 (0.4)	>0.999	2 (0.2)	0 (0.0)	0.488
Previous cerebrovascular accident	10 (4.6)	15 (6.3)	0.551	43 (3.8)	35 (3.2)	0.475
Clinical presentation						
STEMI	96 (44.2)	100 (42.2)	0.730	638 (56.4)	621 (55.9)	0.858
NSTEMI	121 (55.8)	137 (57.8)	0.730	494 (43.6)	490 (44.1)	0.858
Left ventricular ejection fraction <40%	18 (8.3)	10 (4.2)	0.120	85 (7.5)	83 (7.5)	>0.999
Access site						
Radial	122 (56.2)	131 (55.3)	0.914	544 (48.1)	555 (50.0)	0.391
Femoral	90 (41.5)	103 (43.5)	0.740	577 (51.0)	541 (48.7)	0.300
Glycoprotein IIb–IIA inhibitor	43 (19.8)	43 (18.1)	0.738	279 (24.6)	279 (25.1)	0.817
Infarct-related artery (culprit)						
Left main	4 (1.8)	4 (1.7)	>0.999	17 (1.5)	20 (1.8)	0.697
Left anterior descending	119 (54.8)	110 (46.4)	0.089	566 (50.0)	524 (47.2)	0.193
Left circumflex	31 (14.3)	47 (19.8)	0.150	171 (15.1)	217 (19.5)	**0** **.** **007**
Right coronary	63 (29.0)	76 (32.1)	0.549	377 (33.3)	348 (31.3)	0.338
Number of treated vessels, mean (SD)	1.3 (0.6)	1.3 (0.5)	0.469	1.3 (0.6)	1.4 (0.6)	0.093
Multivessel treatment						
2 vessels	44 (20.3)	64 (27.0)	0.116	256 (22.6)	258 (23.2)	0.770
3 vessels	11 (5.1)	15 (6.3)	0.708	60 (5.3)	46 (4.1)	0.232
Number of stents for the infarct-related artery, mean (SD), No	1.2 (0.4)	1.2 (0.4)	0.458	1.2 (0.5)	1.2 (0.5)	0.646
Total stent length of the infarct-related artery, mm (SD)	32.3 (14.1)	31.2 (15.3)	0.414	29.3 (13.0)	29.2 (13.4)	0.837
Stent diameter of the infarct-related artery, mm (SD)	3.1 (0.4)	3.0 (0.4)	0.481	3.2 (0.4)	3.2 (0.5)	0.937
Optical coherence tomography	3 (1.4)	6 (2.5)	0.581	44 (3.9)	29 (2.6)	0.123
Intravascular ultrasonography	50 (23.0)	50 (21.1)	0.714	283 (25.0)	257 (23.1)	0.362

NSTEMI, non-ST-segment elevation myocardial infarction; STEMI, ST-segment elevation myocardial infarction.

The body mass index is the weight in kilograms divided by the square of the height in meters. Impaired renal function was defined as an estimated glomerular filtration rate of less than 60 ml/min per 1.73 m^2^ of body surface area at presentation.

Bold type *p* value, there are statistically significant differences at the significance level of 5%.

### Outcomes by sex

**Figure 1:**
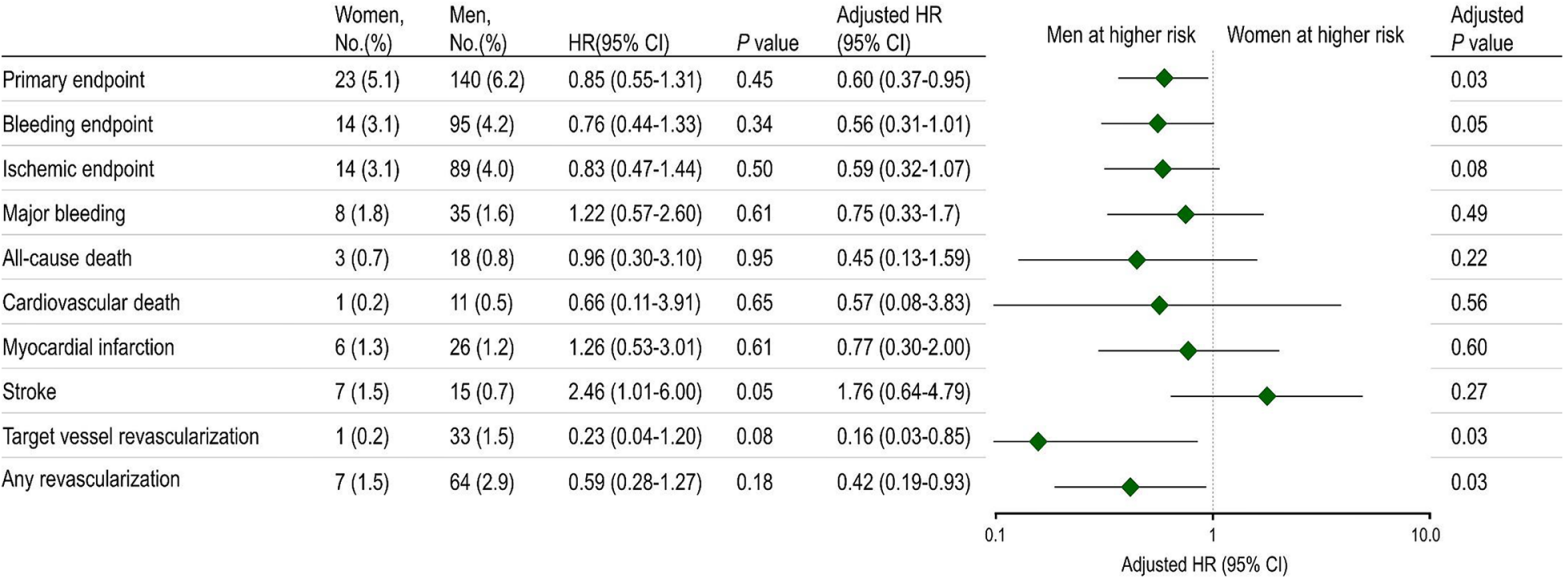
Primary endpoints, bleeding, and ischemic events by sex at 12 months after randomization.

Figure 1 presents the incidence of the primary endpoint at 12 months. There were no statistically significant differences between sexes in the primary endpoint incidence, consisting of cardiovascular death, MI, stroke, and BARC bleeding type 2 or greater. However, following multivariable adjustment, women were observed to have a lower risk of the primary endpoint than men. Although the bleeding endpoint numerically favored lower in women, no statistically significant differences were noted between women and men. The ischemic endpoint also demonstrated a tendency to be lower in women than men. Women exhibited lower rates of target vessel revascularization and any revascularization than men.

### Outcomes by sex and the randomized treatment group

**Figure 2:**
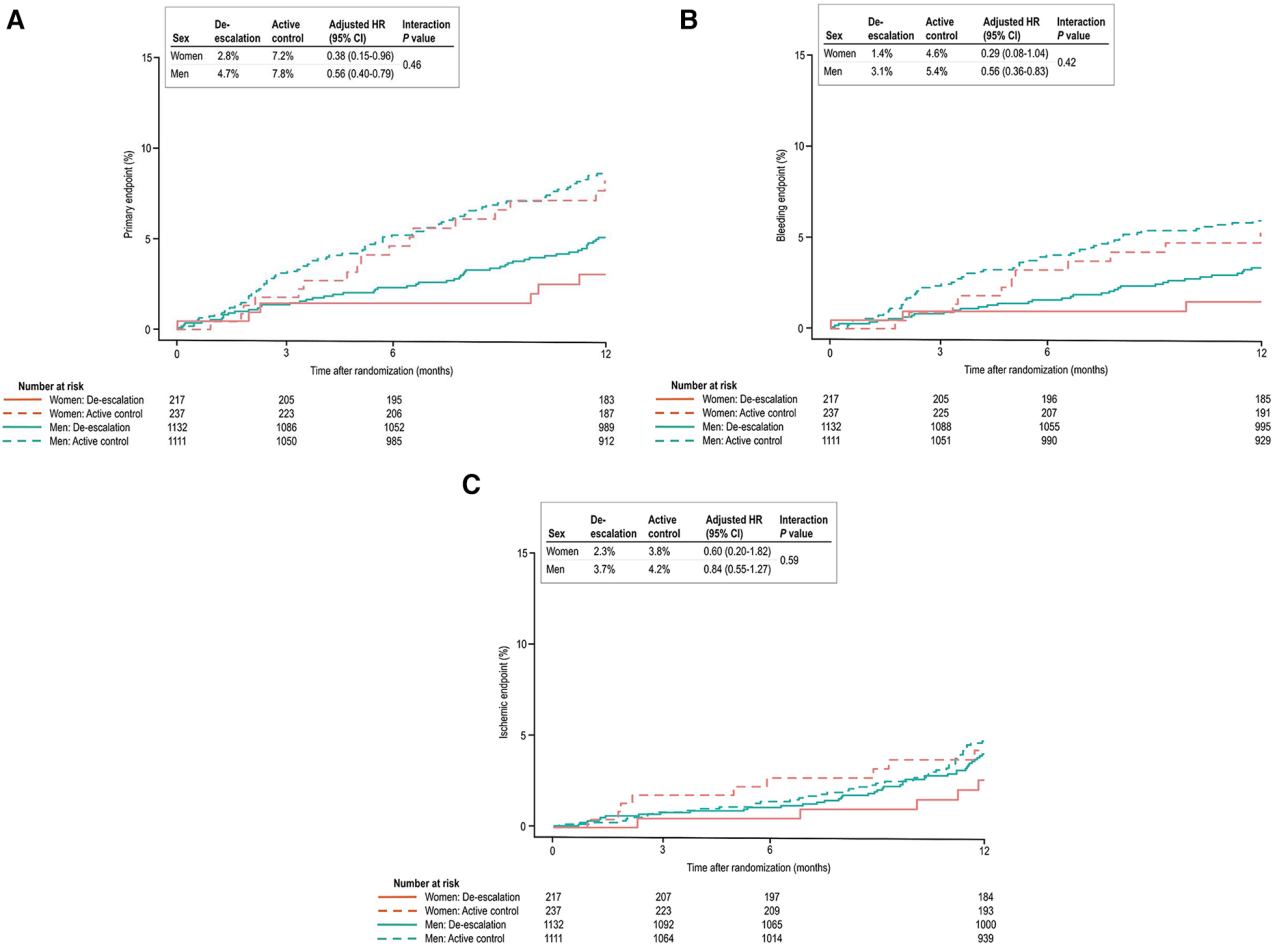
(**A**) Kaplan–Meier estimates and adjusted HRs for the primary endpoint. In both sexes, de-escalation was associated with a lower risk of the primary composite endpoint. (**B**) In bleeding events, the de-escalation group of men exhibited a lower risk of BARC bleeding type 2 or greater. However, in women, these findings did not reach statistical significance, but a trend of lower bleeding risk in the de-escalation group was observed. (**C**) In ischemic events, both sexes were comparable at 12 months after randomization.

Table 3 presents the clinical outcomes by sex and the randomized treatment group at 1 year after randomization. In both sexes, the de-escalation strategy was linked to a decreased risk of the primary composite endpoint. Specifically, within women [6 patients (2.8%) vs. 17 patients (7.2%); adjusted HR, 0.38; 95% CI, 0.15–0.95; *P* = 0.039] and in men [53 patients (4.7%) vs. 87 patients (7.8%); adjusted HR, 0.56; 95% CI, 0.40–0.79; *P* = 0.001], the de-escalation group exhibited a reduced risk of the primary endpoint, with no significant interaction between the randomized treatment group and sex (*P* for interaction = 0.462) (Figure 2A).

**Table 3:** Clinical outcomes by sex and randomized treatment assignment at 12 months after randomization.

	Women (*n* = 454), No. (%)^a^				Men (*n* = 2,243), No. (%)^a^				*P* for interaction^c^
	De-escalation (*n* = 217)	Active control (*n* = 237)	Adjusted HR (95% CI)^b^	*P*-value	De-escalation (*n* = 1,132)	Active control (*n* = 1,111)	Adjusted HR (95% CI)^b^	*P*-value	
Primary endpoint	6 (2.8)	17 (7.2)	0.38 (0.15–0.95)	**0**.**039**	53 (4.7)	87 (7.8)	0.56 (0.40–0.79)	**0**.**001**	0.462
Bleeding endpoint	3 (1.4)	11 (4.6)	0.29 (0.08–1.04)	0.057	35 (3.1)	60 (5.4)	0.55 (0.36–0.83)	**0**.**005**	0.423
Ischemic endpoint	5 (2.3)	9 (3.8)	0.60 (0.20–1.82)	0.367	42 (3.7)	47 (4.2)	0.84 (0.55–1.27)	0.399	0.586
Major bleeding (BARC 3 or 5)	2 (0.9)	6 (2.5)	0.29 (0.05–1.52)	0.143	13 (1.1)	22 (2.0)	0.57 (0.29–1.12)	0.104	0.703
All-cause death	1 (0.5)	2 (0.8)	0.80 (0.08–8.26)	0.851	10 (0.9)	8 (0.7)	1.16 (0.46–2.96)	0.754	0.584
Cardiovascular death	0	1 (0.4)	0.30 (0.003–26.01)	0.596	6 (0.5)	5 (0.5)	1.15 (0.35–3.78)	0.820	0.456
Myocardial infarction	3 (1.4)	3 (1.3)	1.41 (0.24–8.17)	0.702	9 (0.8)	17 (1.5)	0.51 (0.23–1.15)	0.106	0.407
Stroke	2 (0.9)	5 (2.1)	0.39 (0.07–2.12)	0.276	7 (0.6)	8 (0.7)	0.85 (0.31–2.35)	0.750	0.674
Target lesion revascularization	0	0	—	—	14 (1.2)	9 (0.8)	1.46 (0.63–3.37)	0.377	0.862
Target vessel revascularization	0	1 (0.4)	0.79 (0.01–72.75)	0.919	17 (1.5)	16 (1.4)	1.00 (0.51–1.99)	0.995	0.522
Any revascularization	3 (1.4)	4 (1.7)	1.01 (0.20–4.96)	0.993	29 (2.6)	35 (3.2)	0.78 (0.48–1.29)	0.335	0.978
Stent thrombosis	0	0	—	—	3 (0.3)	3 (0.3)	0.92 (0.18–4.67)	0.922	0.998

The primary endpoint is defined as a composite of cardiovascular death, myocardial infarction, stroke, or BARC bleeding type 2, 3, or 5. The bleeding endpoint is a composite of BARC bleeding types 2, 3, or 5. The ischemic endpoint is a composite of cardiovascular death, myocardial infarction, stroke, any revascularization, or stent thrombosis.

^a^ The percentages represent Kaplan–Meier rates at 12 months after randomization.

^b^ The model is adjusted for age, body mass index, hypertension, diabetes, current smoking, impaired renal function, access site, glycoprotein IIb–IIIa inhibitor, number of stents, total stent length, and mean stent diameter.

^c^ Interaction test between randomized treatment assignment and sex after model adjustment.

Bold type *p* value, there are statistically significant differences at the significance level of 5%.

**Figure 3:**
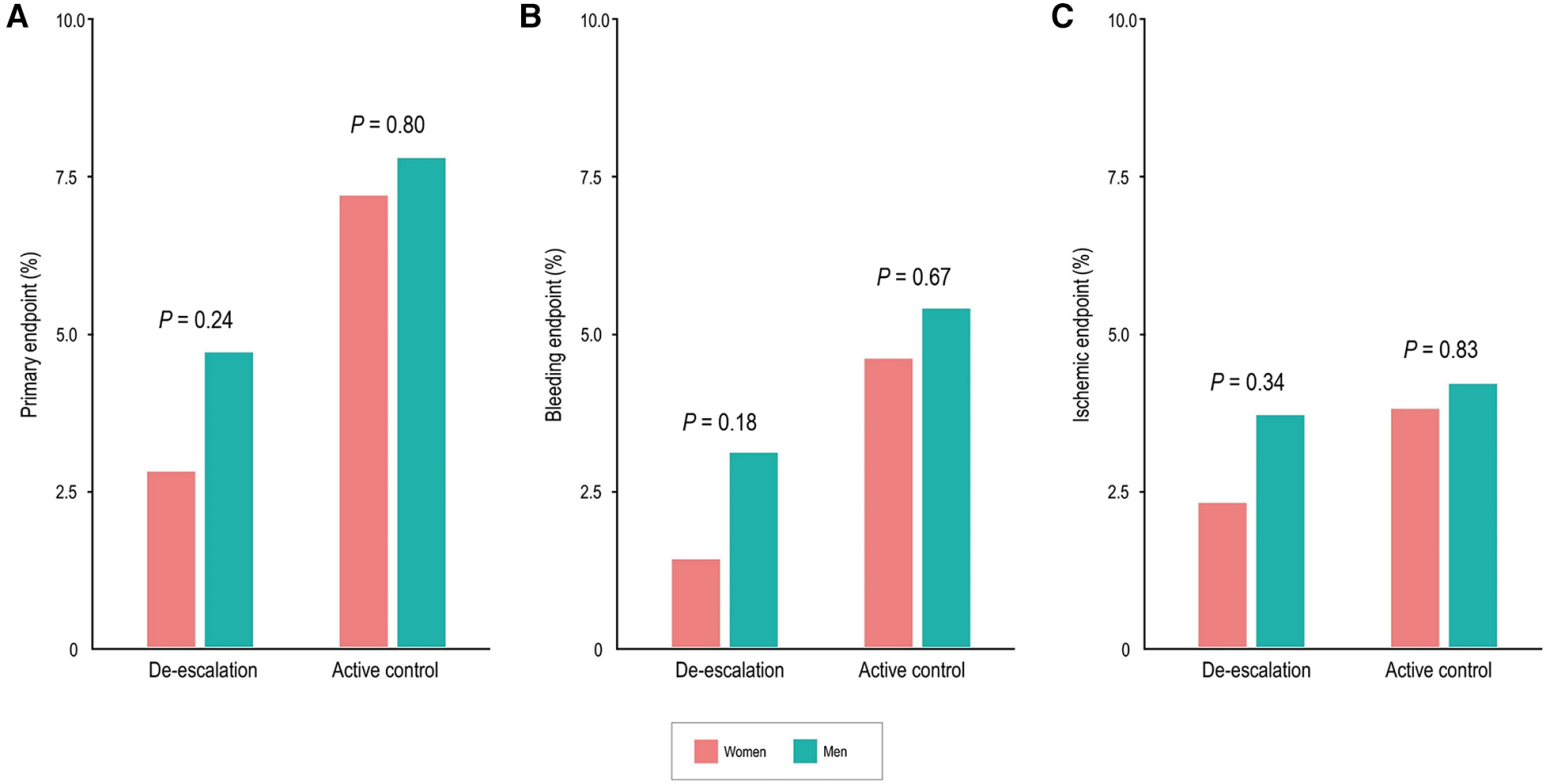
Sex differences of clinical outcomes in the randomized treatment group at 12 months. Women had comparable rates of the primary endpoint (**A**), bleeding events (BARC type 2, 3, or 5) (**B**), and ischemic events (**C**) compared with men.

In the de-escalation group of men, the risk of BARC bleeding type 2 or greater was lower [35 patients (3.1%) vs. 60 patients (5.4%); adjusted HR, 0.55; 95% CI, 0.36–0.83; *P* = 0.005). However, in women, these findings were not significant, but there was a trend of lower bleeding risk in the de-escalation group of women [3 patients (1.4%) vs. 11 patients (4.6%); adjusted HR, 0.29; 95% CI, 0.08–1.04; *P* = 0.057). No significant interaction between the randomized treatment group and sex was observed (*P* for interaction = 0.423) (Figure 2B), and the ischemic endpoint was comparable (Figure 2C). In the randomized treatment group, the rates of clinical outcomes were similar in both women and men (Figure 3).

## Discussion

In this *post-hoc* analysis of the TALOS-AMI study, notable differences in baseline characteristics were observed between the sexes. Women exhibited a significantly older age and a higher prevalence of risk factors compared to men. Among stabilized patients with AMI after PCI with DES, women showed a lower incidence of the primary composite endpoint than men. Furthermore, the de-escalation strategy, when compared to the active control group, significantly reduced the risk of the primary composite endpoint in both sexes. The bleeding risk was significantly lower in the de-escalation group of men than in the active control of men. Although women in the de-escalation group showed a lower risk, it did not reach statistical significance due to a lower event rate. The ischemic endpoint was similar between the randomized treatment groups and across sexes. Compared to men, women experienced comparable rates of clinical outcomes within the randomized treatment group.

Sex-based studies consistently reported that women have higher incidences of both ischemic and bleeding events after PCI, including in patients with AMI (3–6, 9, 10). Furthermore, women tend to experience higher in-hospital or short-term mortality when undergoing PCI for AMI (11–14). This finding is attributed to differences in baseline comorbidities, particularly older age, rather than biological factors, with a higher prevalence of diabetes, renal insufficiency, and increased platelet reactivity in older women (15). In a previous real-world study, elderly ACS patients (>65 years old) carrying CYP2C19 loss-of-function alleles had a higher incidence of bleeding and similar rates of ischemic events (16). While previous research has indicated that women are at higher risk of ischemic and bleeding events following PCI compared to men (3–6), it remains uncertain whether women face an increased risk of these events during the stabilized period following AMI and PCI with DES. Given that this study included both men and women with a stable clinical status 1 month after AMI, it is likely that the sex differences observed in the acute phase of AMI, as reported in prior studies, were not evident.

To our knowledge, the TALOS-AMI study is the first large-scale randomized, controlled study to investigate the efficacy and safety of an unguided de-escalation strategy, transitioning from ticagrelor to clopidogrel one month after AMI in stabilized patients without major ischemic or bleeding events during the first month following PCI. This study reveals that the adoption of an unguided de-escalation DAPT strategy by switching from ticagrelor to clopidogrel after 1 month of AMI is not only non-inferior but even superior to the standard DAPT strategy based on ticagrelor (2). These results hold significance as unguided de-escalation of DAPT from potent P2Y_12_ inhibitors to clopidogrel after the acute phase of AMI is a common practice in clinical settings (17–19). While platelet function testing-guided strategies for de-escalation can assess platelet reactivity during treatment, they suffer from considerable inter-assay variability, and the optimal timing remains undetermined, limiting their clinical applicability. The unguided de-escalation strategy was associated with a 45% lower risk of net clinical benefits compared to the ticagrelor-based DAPT strategy for the subsequent 11 months. The absolute risk reduction was 3.6%, primarily attributed to a significant decrease in bleeding risk (2). However, despite previous studies showing that women have an increased bleeding risk compared to men after PCI (3–5), our analysis did not align with these findings. We did not observe a significant increase in bleeding risk among women in the chronic phase after PCI with DES. In fact, men tended to have a higher bleeding risk.

In patients with AMI, current guidelines continue to recommend the continuation DAPT with a potent P2Y12 inhibitor for at least 12 months (20). In this context, the study offers clinical evidence supporting the practically and feasibility of an unguided DAPT de-escalation strategy after the acute phase of AMI. This approach is considered more realistic and attainable compared to a guided de-escalation strategy or transition to monotherapy with a potent P2Y12 inhibitor, and it demonstrated effectiveness in both sexes. While there may be variations in the relative benefit between sexes, even if there is a similar reduction in the relative risk of bleeding, it is expected to enhance the advantages of the de-escalation strategy for women. In fact, the absolute risk reduction for BARC type 2, 3, or 5 bleeding was numerically higher in women compared to men. This finding is supported by the indication of a potential late bleeding advantage in women who underwent the de-escalation strategy. Overall, the results of this analysis suggest that an early switch to clopidogrel after PCI is preferable for both women and men, especially when they are on a ticagrelor-based DAPT regimen. Recently, the consensus document from the Academic Research Consortium recommends modulation of antiplatelet therapy for patients undergoing PCI, with the optimal intensity of platelet inhibition (de-escalation or escalation) varying according to the stage and presentation of coronary artery disease and individual patient factors (21). In the future, it is expected to improve antiplatelet therapy outcomes by considering sex-specific differences. Tailoring treatments based on these variations aims to provide more effective and personalized care for individuals of all genders, promoting better clinical results.

This study has several limitations that warrant consideration. It was a *post-hoc* analysis; the findings should be regarded as suggestive and require validation in future studies. The randomization process did not involve sex stratification and failed to account for multiplicity, thereby elevating the risk of type I error. Furthermore, the women subgroup was relatively small, and notable differences existed in baseline risk levels between the sexes. Imbalances in patient characteristics persisted among the sex-specific treatment groups, including variables such as diabetes, hypertension, and impaired renal function. As randomization lacked stratification based on sex, complete elimination of residual confounding remains challenging even after multivariable adjustment for baseline differences. In addition, due to the limited number of women in the study, both sex-specific subgroups lacked sufficient statistical power to draw definitive conclusions regarding the effect of de-escalation vs. active control on the primary composite and bleeding endpoint. The study findings are confined to a population that underwent PCI during the stabilized period after DES implantation, potentially limiting their applicability to the broader patient population undergoing PCI. Moreover, the analyses only included patients who had successfully undergone PCI with DES and experienced no clinical events within one month of the procedure while continuing to receive DAPT. Finally, given that this study was conducted on East Asian patients, the presence of the East Asian paradox suggests that caution should be exercised in generalizing the results. Nonetheless, in a recent meta-analysis, the substantiating evidence and the safety profiles of various strategies aimed at reducing bleeding through antiplatelet treatment regimens may be notably influenced by ethnic factors, necessitating consideration in clinical application (22).

## Conclusions

In conclusion, this *post-hoc* analysis of the TALOS-AMI randomized clinical study revealed that stabilized patients with AMI after PCI with DES experienced a lower incidence of the primary endpoint among women compared to men. The advantages of the unguided de-escalation strategy from ticagrelor to clopidogrel were generally comparable in both women and men.

## Data Availability

The datasets presented in this study can be found in online repositories. The names of the repository/repositories and accession number(s) can be found below: The datasets generated and/or analyzed during the current study are not publicly available but are available from the corresponding author on reasonable request.
